# Key gait findings for diagnosing three syndromic categories of dynamic instability in patients with balance disorders

**DOI:** 10.1007/s00415-020-09901-5

**Published:** 2020-05-27

**Authors:** Roman Schniepp, Ken Möhwald, Max Wuehr

**Affiliations:** 1 Department of Neurology, Ludwig-Maximilian University of Munich, Munich, Bavaria Germany; 2 German Center for Vertigo and Balance Disorders (DSGZ), Ludwig-Maximilian University of Munich, Munich, Bavaria Germany

**Keywords:** Instrumented gait analysis, Multi-condition gait examination, Pattern recognition, Ataxia, Hypokinesia, Dementia

## Abstract

With the emergence of affordable, clinical-orientated gait analysis techniques, clinicians may benefit from a general understanding of quantitative gait analysis procedures and their clinical applications. This article provides an overview of the potential of a quantitative gait analysis for decision support in three clinically relevant scenarios of early stage gait disorders: scenario I: gait ataxia and unsteadiness; scenario II: hypokinesia and slow gait; scenario III: apparently normal gait with a specific fall tendency in complex mobility situations. In a first part, we justify the advantages of standardized data collection and analysis procedures including data normalization and dimensionality reduction techniques that facilitate clinical interpretability of instrument-based gait profiles. We then outline typical patterns of pathological gait and their modulation during different walking conditions (variation of speed, sensory perturbation, and dual tasking) and highlight key aspects that are particularly helpful to support and guide clinical decision-making.

## Introduction

Gait instability is prevalent in patients with balance problems, vertigo, and dizziness, and is associated with adverse health outcomes. Depending on the underlying disease entity, the risk of falling and consequent injuries is markedly increased [20]. Falls are closely linked to morbidity and often eventuate in a reduction of the quality of life. Thus, the clinical assessment of patients with balance disorders should include procedures that focus on individual gait function. Clinical observation of gait and simple clinical balance tasks (e.g. Romberg’s test) are well established but limited in certain respects. Accordingly, outcomes and interpretation from clinical assessment are highly dependent on the examiner’s experience and clinical background and show a low inter-rater reliability. This is particularly problematic in patients with balance problems and dizziness that are often examined by physicians from different disciplines. Moreover, gait impairments associated with peripheral or central sensory disorders are often subtle and thus difficult to detect by the clinician’s eye.

In this context, quantitative, instrument-based gait analysis is a promising tool to capture and accurately assess gait function. Clinical approaches with a justifiable trade-off between the clinical benefit and infrastructural resources have been recently established. Central for clinical implementation of these techniques is application of standardized protocols for the recording, the analysis, and the interpretation of clinical gait profiles. Clinical experience and evidence from several studies emphasize that clinical gait examination should in particular assess patients’ gait function during different walking conditions [5, 8, 15]. Accordingly, it has been shown that walking at non-habitual speeds [28, 32], perturbation of sensory feedback [33], and cognitive and motor dual-task paradigms [2, 3] are suitable to unmask subtle gait impairments in geriatric and neurological patients. Multi-condition gait assessment thereby facilitates a superior diagnostic accuracy [1].

This article provides an overview of typical alterations of spatial and temporal gait features related to three basic syndromic scenarios: (1) gait ataxia and unsteadiness of gait with fall risk; (2) hypokinesia and slow gait; (3) fall tendency in complex situations (apparently with normal walking behavior). A special emphasis is placed on the relevance of gait findings for supporting clinical differential diagnosis.

## Multi-condition gait assessment and data visualization

The clinical gait profiles, which are discussed in the subsequent paragraphs were recorded on a pressure-sensitive gait carpet (GAITRite^®^, CIR System, Franklin, NJ, USA). A multi-condition assessment protocol with walking at self-chosen walking speed (PWS), at slow speed (SS), at maximally fast speed (MS), with reclination of the head (HR), with eyes closed (EC), with performance of a serial seven dual task (DTC), with performance of a verbal fluency dual task (DTS), and while carrying an empty tray (DTM) was performed as described elsewhere [15, 22].

**Fig. 1:**
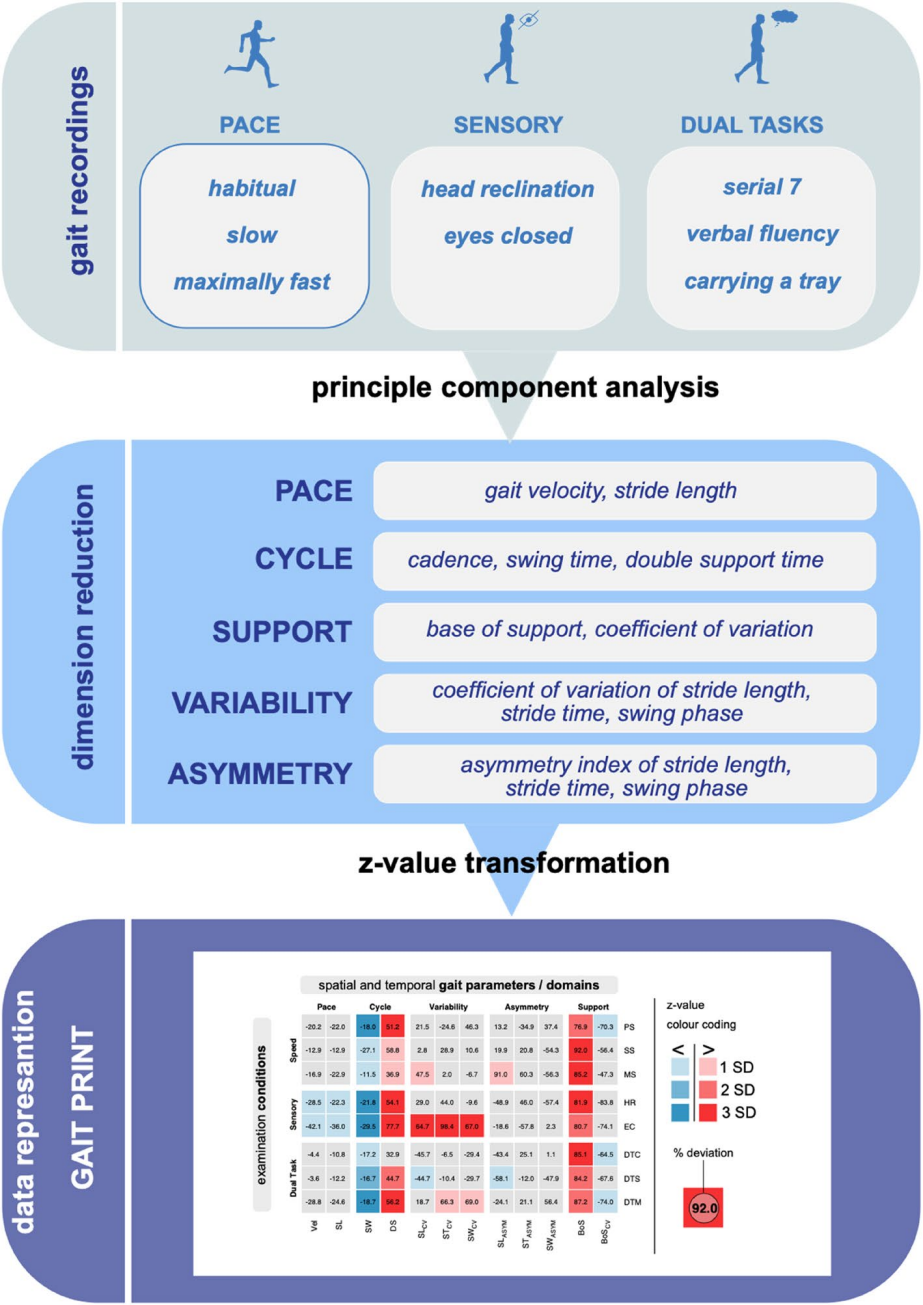
Gait data acquisition, preprocessing, and analysis. Work flow of gait data processing. Gait performance is recorded using a *multi-condition* examination protocol with walking speed variation, perturbation of sensory feedback, and dual-task paradigms. Spatial and temporal gait parameters are categorized to five distinct gait domains based on *principle component analysis*. Each parameter is normalized via *z-value transformation* (reference to an age- and gender-matched control group). Percentual difference from group mean is presented numerically and *z*-values are illustrated by colour coding. *PS* preferred walking, *SS* slow walking, *MS* maximally fast walking, *HR* walking with head reclination, *EC* walking with eyes closed, *DTC* walking with a serial 7 dual task, *DTS* walking with a verbal fluency dual task, *DTM* walking while carrying a tray, *Vel* gait velocity, *SL* stride length, *SW* swing phase, *DS* double support phase, *SL*_*CV*_ coefficient of variation of stride length, *ST*_*CV*_ coefficient of variation of stride time, *SW*_*CV*_ coefficient of variation of swing phase, *SL*_*ASYM*_ asymmetry of stride length, *ST*_*ASYM*_ asymmetry of stride time, *SW*_*ASYM*_ asymmetry of swing phase, *BoS* base of support, *BoS*_*CV*_ coefficient of variation of base of support

To handle the data complexity related to multi-condition gait recordings, two principle data analysis steps are particularly helpful. In a first step, by means of a principle component analysis (an established dimensionality reduction technique for complex datasets) [13], gait parameters are arranged according to five distinct macro variables (gait domains) that characterize independent functional domains of walking, namely pace, cycle, variability, asymmetry, and support. In a second step, each quantified gait parameter becomes normalized in terms of a *z*-value transformation based on an age- and gender-matched healthy control group (*n* = 396). The healthy control group was collected by direct recruitment and covers the age spectrum from 18 to 99 years. The resulting data are arranged in a color-coded data matrix that at first glance provides a rapid overview on the individual patient’s gait performance under various challenging conditions in comparison to age- and gender-matched healthy reference performance (see Fig. 1).

## Scenario I: gait ataxia and unsteadiness

**Fig. 2:**
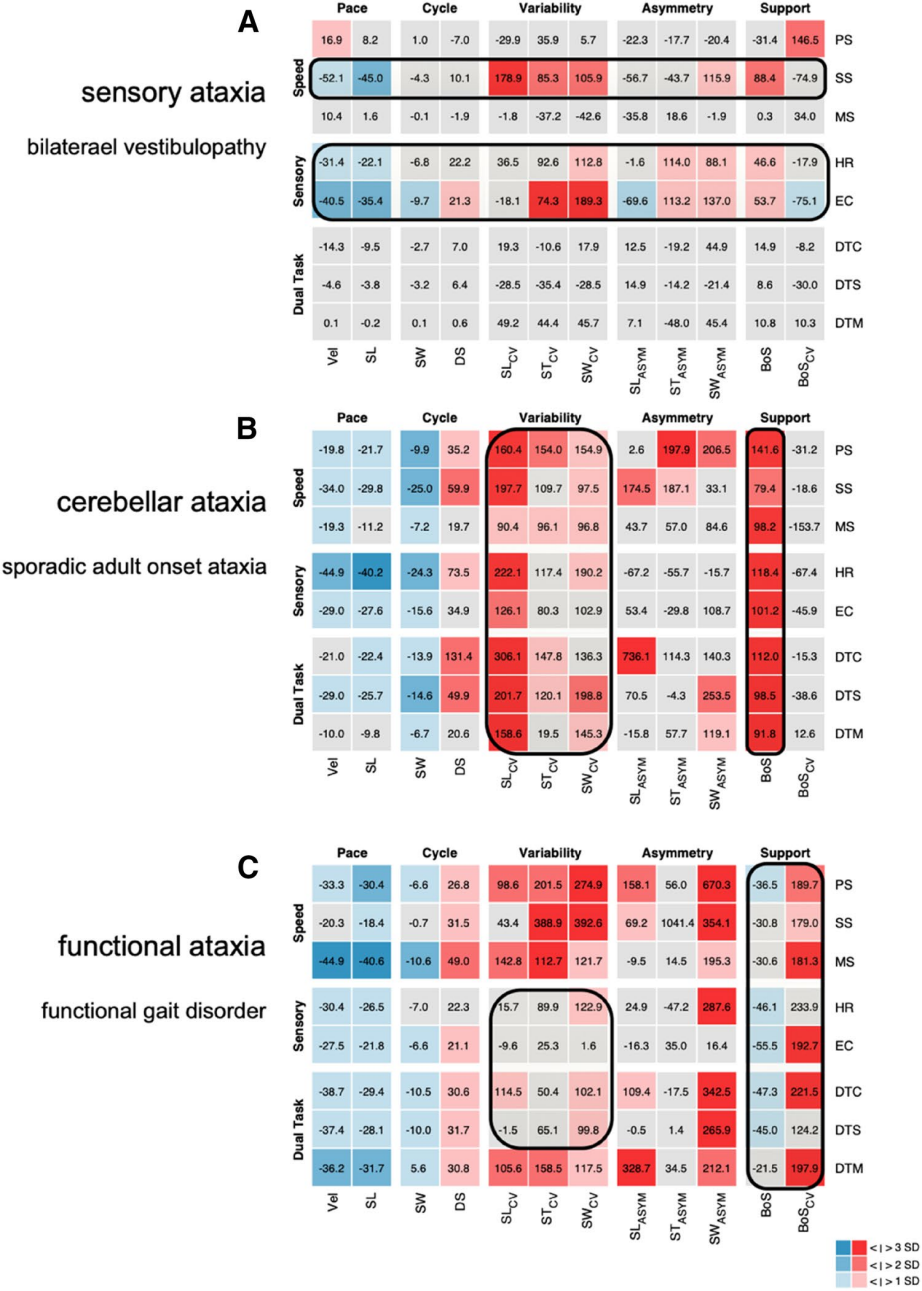
Scenario: ‘gait ataxia/unsteadiness’. Three *z*-value matrixes of multi-condition gait assessment in patients with ‘gait ataxia/unsteadiness’. **a** represents the matrix of a 56-year-old patient with bilateral vestibulopathy. This gait disorder is characterized by condition-dependent alterations of pace, support, and variability parameters. Alterations are present during conditions of sensory pertubation and slow walking. **b** represents the matrix of a 67-year-old patient with sporadic adult onset cerebellar ataxia. The hallmark of cerebellar gait ataxia is an increased gait variability with a broadened base of support. These features are consistently present throughout all examination conditions. **c** represents the matrix of a 45-year-old patient with a functional ataxia. Gait variability is strongly increased and pace markedly reduced. In contrast, base of support is narrow to normal. The variability of the base of support is particularily increased, indicating a scissor-type stepping pattern. The gait pattern is less consistent and shows paradox improvements during the performance of sensory perturbation tasks or cognitive dual tasks. *PS* preferred walking, *SS* slow walking, *MS* maximally fast walking, *HR* walking with head reclination, *EC* walking with eyes closed, *DTC* walking with a serial 7 dual task, *DTS* walking with a verbal fluency dual task, *DTM* walking while carrying a tray, *Vel* gait velocity, *SL* stride length, *SW* swing phase, *DS* double support phase, *SL*_*CV*_ coefficient of variation of stride length, *ST*_*CV*_ coefficient of variation of stride time, *SW*_*CV*_ coefficient of variation of swing phase, *SL*_*ASYM*_ asymmetry of stride length, *ST*_*ASYM*_ asymmetry of stride time, *SW*_*ASYM*_ asymmetry of swing phase, *BoS* base of support, *BoS*_*CV*_ coefficient of variation of base of support

The hallmarks of the gait impairments in patients with sensory deficits, cerebellar disorders, or functional ataxia comprise distinct alterations in variability and support domains of walking. Accordingly, the spatial and temporal variability of stepping is typically increased in these cohorts [11]—a gait alteration that is directly related to an impaired dynamic stability and an increased risk of falls in patients with vestibular [25] and cerebellar ataxia [24, 29]. Furthermore, the severity of ataxia symptoms has been shown to correlate with the magnitude of gait variability in patients with cerebellar ataxia [10, 24]. Patients with a marked gait instability typically exhibit a broadening of the base of support which can be interpreted as a compensatory strategy to ensure dynamic balance while walking. This compensatory behavior is particularily characteristic for patients with cerebellar disorders [17], and can be also observed but less pronounced in patients with vestibular disorders [23]. In contrast, patients with functional ataxia frequently walk with a narrow or normal base of support despite their apparently increased gait variability. The characteristic discordancy between the seemingly instable walking performance and the absence of compensatory stabilization strategies in functional gait ataxia is especially useful to differentiate this gait disorder from cerebellar ataxia (see Fig. 2).

Walking pace in patients with cerebellar and sensory ataxia (particularly at the initial phase of the disease) is typically preserved or only slightly decreased. This observation can be explained by the fact that sensory integration for gait stabilization is speed dependent and less essential during fast locomotion [9]. Accordingly, patients with peripheral or central sensory integration problems strive for a faster, automated locomotion pattern that primarily relies on spinal locomotor automatisms [6]. Patients with functional ataxia, however, typically show a strong reduction of walking pace, which correlates with the amount of anxiety and fear of falling [21, 27].

The characteristic modulation of ataxic gait impairments during multi-condition gait assessment can further support the differential diagnosis in the context of gait ataxia. Slow walking modes accentuate gait instability in patients with vestibular and cerebellar disorders, in particular in terms of a considerably increased gait variability [25]. As mentioned above, sensory feedback control becomes less important with faster locomotion and sensory ataxic gait pattern accordingly normalizes at moderate to fast walking. Withdrawal of visual feedback has stronger deteriorating effects on gait instability in patients with sensory deficits compared to patients with cerebellar ataxia. In contrast, patients with functional ataxia show a paradoxical improvement during fast walking modes [22] and during cognitive dual-task conditions, presumably due to a distraction of the excessive attentional focus on balance adjustments in these patients [31].

## Scenario II: hypokinesia and slow gait

**Fig. 3:**
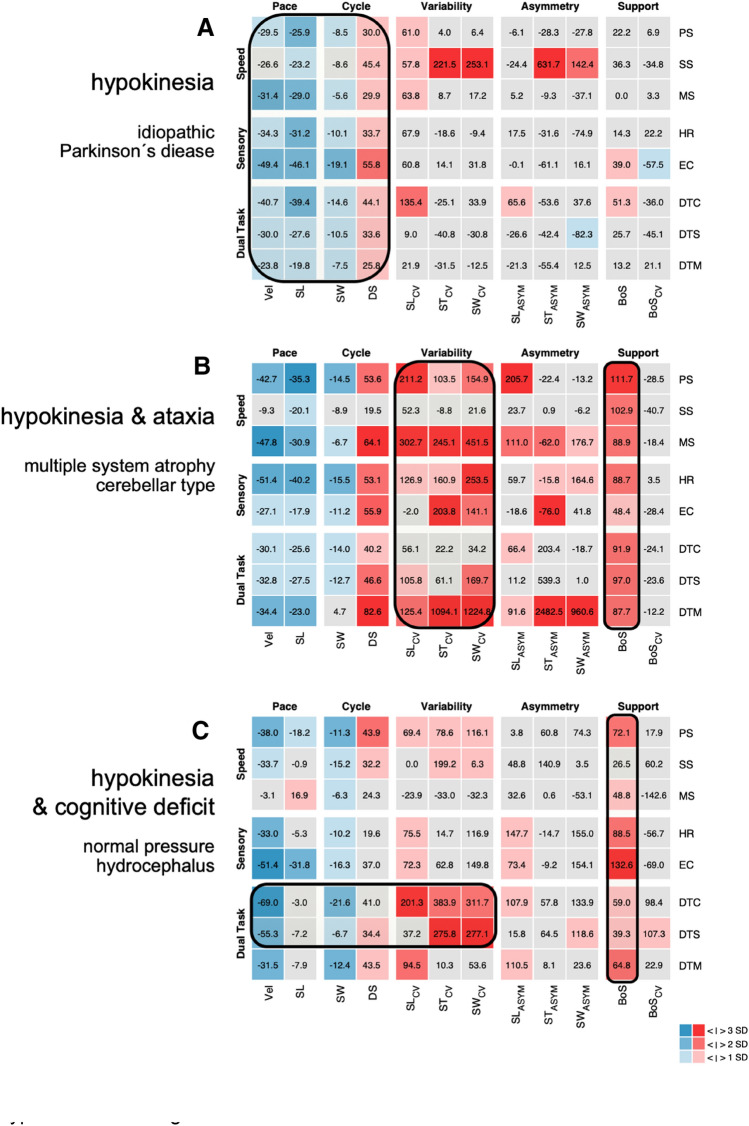
Scenario: ‘hypokinesia/slow gait’. Three *z*-value matrixes of multi-condition gait assessment of patients with ‘hypokinesia/slow gait’. **a** represents the matrix of a 67-year-old patient with idiopathic Parkinson’s disease. Pace and cycle parameters show persistent alterations over all conditions. The base of support is normal in the early stage of the disease. **b** represents the matrix of a 63-year-old patient with multiple system atrophy and cerebellar degenaration. Besides the alteration of pace and cycle parameters, variability and the base of support are particularily increased in accordance to an ataxic component. **c** represents the matrix of a 75-year-old patient with a normal pressure hydrocpehalus. It is characterized by a reduction of pace and cycle parameters with increased base of support. During cognitive dual task, there is a further deterioration of gait with a reduction of pace and an increase of gait variability. *PS* preferred walking, *SS* slow walking, *MS* maximally fast walking, *HR* walking with head reclination, *EC* walking with eyes closed, *DTC* walking with a serial 7 dual task, *DTS* walking with a verbal fluency dual task, *DTM* walking while carrying a tray, *Vel* gait velocity, *SL* stride length, *SW* swing phase, *DS* double support phase, *SL*_*CV*_ coefficient of variation of stride length, *ST*_*CV*_ coefficient of variation of stride time, *SW*_*CV*_ coefficient of variation of swing phase, *SL*_*ASYM*_ asymmetry of stride length, *ST*_*ASYM*_ asymmetry of stride time, *SW*_*ASYM*_ asymmetry of swing phase, *BoS* base of support, *BoS*_*CV*_ coefficient of variation of base of support

Hypokinetic gait patterns can be observed in patients with Parkinsonism, patients with subcortical vascular encephalopathy (SAE), and patients with normal pressure hydrocephalus (NPH). The general identification of hypokinetic gait is not challenging for experienced neurologists. Typical features of gait impairments related to this syndromic category include a reduction of stepping pace, a reduced stride length, and reduced foot clearance during swing phases. Variability measures can be increased, especially in the late course of disease or when freezing-of-gait episodes are present [19]. Patients with a cerebellar type of multiple system atrophy (MSA-c) can initially exhibit a dominant ataxic phenotype, but typically develop hypokinetic gait alterations within the course of the first two years of disease [12]. The base of support in early stage idiopathic Parkinson’s disease (IPS) is typically normal, in contrast to atypical forms of hypokinetic gait as NPH or SAE [30]. Thus, the quantification of the base of support is crucial for the differential diagnosis of hypokinetic gait disorders (Fig. 3).

In hypokinetic gait disorders, alterations of gait parameters typically persist during all examination conditions. Patients with SAE or NPH show a further decline of walking performance during cognitive dual task [4, 26, 30]—a gait assessment condition that is particularly suited to examine the motor–cognitive interaction during walking. In hypokinetic gait disorders, dual tasking commonly results in a further decrease of pace and cycle parameters. Additionally, interruption of walking during the execution of the cognitive task (e.g. “stop walking when talking”) is a typical finding that can be interpreted as a motor symptom that results from a primary underling cognitive deficit [14]. Freezing of gait is a clinical phenomenon characterized by brief episodes of a discontinuation of stepping (typically during the toe-off phase prior to the swing phase). Freezing of gait is predominantly present in patients with basal ganglia disorders, but can also in rare cases be observed in patients with SAE or NPH [18].

## Scenario III: fall tendency in complex situations

**Fig. 4:**
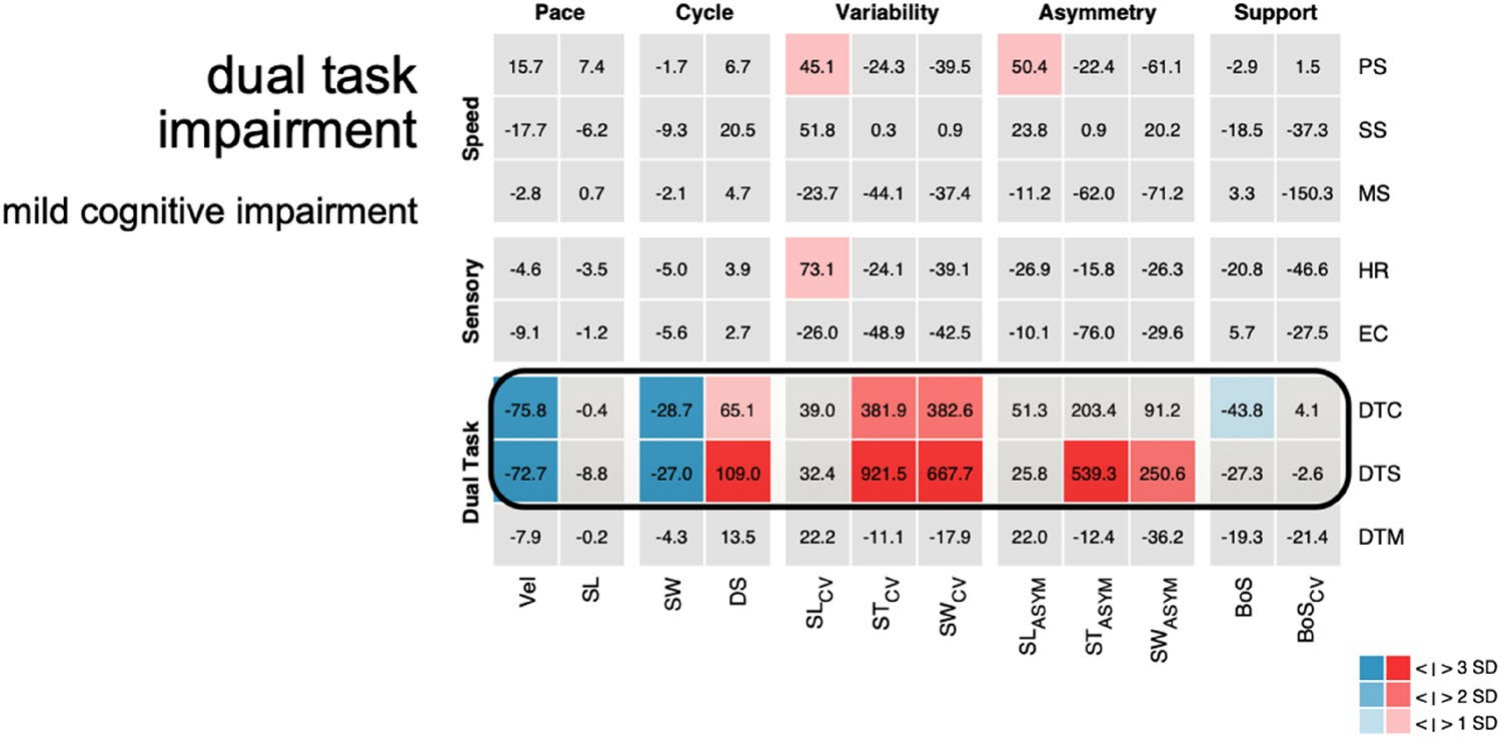
Scenario: ‘fall tendency in complex mobility situations’. *Z*-value matrix of multi-condition gait assessment of a patient with mild cognitive impairment. The matrix represents the walking behavior of a 64-year-old patient with mild cognitive impairment. No obvious gait alterations are present during single-task walking and sensory perturbation. However, cognitive dual-task conditions disculose the actual underlying gait impairment. Accordingly, the complex motor–cognitive interaction results in a reduction of pace and cycle parameters with a relevant increase of variability. The gait pattern is highly instable and prone to falls. Episodes of brief interruptions of locomotion during the execution of the cognitive dual task are frequent (e.g. ‘stop walking when talking’ phenomen). In contrast, gait performance during motor–motor dual task is normal. *PS* preferred walking, *SS* slow walking, *MS* maximally fast walking, *HR* walking with head reclination, *EC* walking with eyes closed, *DTC* walking with a serial 7 dual task, *DTS* walking with a verbal fluency dual task, *DTM* walking while carrying a tray, *Vel* gait velocity, *SL* stride length, *SW* swing phase, *DS* double support phase, *SL*_*CV*_ coefficient of variation of stride length, *ST*_*CV*_ coefficient of variation of stride time, *SW*_*CV*_ coefficient of variation of swing phase, *SL*_*ASYM*_ asymmetry of stride length, *ST*_*ASYM*_ asymmetry of stride time, *SW*_*ASYM*_ asymmetry of swing phase, *BoS* base of support, *BoS*_*CV*_ coefficient of variation of base of support

Besides the interaction of sensory feedback with supraspinal and spinal locomotor regions, human postural control also relies on cognitive and attentional capacities. Patients with mild cognitive dysfunctions frequently consult clinical centers for balance problems due to apparent locomotion impairments. They typically report a slowing of gait during real-world mobility and the occurrence of falls in complex mobility situations. The clinical examination of sensory functions and simple motor tasks does not show any significant findings in these patients. Instrument-based gait analysis shows normal gait performance during single-task walking, but typically yields strikingly abnormal findings during cognitive dual-task conditions. Accordingly, walking pace and stride length become considerably decreased [16] and walking becomes highly irregular (Fig. 4). In addition, patients frequently exhibit spontaneous interruptions of walking during the performance the additional cognitive task (so called “stop walking when talking” episodes) [14]. Thus, instrument-based gait analysis including the examination of cognitive dual tasks is particularly helpful to disclose an early, subtle deterioration of motor-cognitive capacity in patient’s mild cognitive impairments [7].

## Summary

Disturbances of mobility and gait are major symptoms of patients with balance disorders, dizziness, or vertigo. They are related to deteriorations of the functional status and to severe health outcomes, such as falls and fall-related morbidity [20]. The clinical application of quantitative, instrument-based gait analysis procedures can help to assess the degree of dynamic gait instability [25] and to predict individual patient’s fall risk [19, 23]. Moreover, quantitative gait assessment has the potential to facilitate differential diagnostic decisions in early stage gait disorders. The clinical applicability of these assessment routines relies on an easy-to-assess form of data representation. In particular, a reduction of spatiotemporal gait parameters to five distinct gait domains and standardized data normalization and visualization techniques can facilitate the trade-off between infrastructural efforts and the clinical usefulness of instrument-based gait analysis. Superior diagnostic validity can be achieved by performing a standardized multi-conditions gait protocol, including walking at non-habitual speeds, perturbations of sensory feedback, and the performance of cognitive dual tasks.
